# Silver nanoparticle-enriched diamond-like carbon implant modification as a mammalian cell compatible surface with antimicrobial properties

**DOI:** 10.1038/srep22849

**Published:** 2016-03-09

**Authors:** Christian Gorzelanny, Ralf Kmeth, Andreas Obermeier, Alexander T. Bauer, Natalia Halter, Katharina Kümpel, Matthias F. Schneider, Achim Wixforth, Hans Gollwitzer, Rainer Burgkart, Bernd Stritzker, Stefan W. Schneider

**Affiliations:** 1 Experimental Dermatology, Department of Dermatology, Medical Faculty Mannheim, Heidelberg University, Mannheim, Germany; 2 Experimental Physics IV, Physics Institute, Augsburg University, Augsburg, Germany; 3 Clinic of Orthopedics and Traumatology, Technical University Munich, 81675 Munich, Germany; 4 Department of Mechanical Engineering, Boston University, Boston, MA 02215, US; 5 Experimental Physics I, Physics Institute, Augsburg University, Augsburg, Germany; 6 Augsburg Center for Innovative Technologies, Augsburg, Germany

## Abstract

The implant-bone interface is the scene of competition between microorganisms and distinct types of tissue cells. In the past, various strategies have been followed to support bony integration and to prevent bacterial implant-associated infections. In the present study we investigated the biological properties of diamond-like carbon (DLC) surfaces containing silver nanoparticles. DLC is a promising material for the modification of medical implants providing high mechanical and chemical stability and a high degree of biocompatibility. DLC surface modifications with varying silver concentrations were generated on medical-grade titanium discs, using plasma immersion ion implantation-induced densification of silver nanoparticle-containing polyvinylpyrrolidone polymer solutions. Immersion of implants in aqueous liquids resulted in a rapid silver release reducing the growth of surface-bound and planktonic *Staphylococcus aureus* and *Staphylococcus epidermidis*. Due to the fast and transient release of silver ions from the modified implants, the surfaces became biocompatible, ensuring growth of mammalian cells. Human endothelial cells retained their cellular differentiation as indicated by the intracellular formation of Weibel-Palade bodies and a high responsiveness towards histamine. Our findings indicate that the integration of silver nanoparticles into DLC prevents bacterial colonization due to a fast initial release of silver ions, facilitating the growth of silver susceptible mammalian cells subsequently.

Nanostructured or soft-compound coated surfaces have been successfully tested for cell engineering processes and proposed as biointerfaces in the past. Deposition of vascular endothelial cell growth factor (VEGF) or nanopattering of the implant surface were shown to promote the growth of particular cell types such as osteoblasts and endothelial cells^1^,^2^. While osteoblasts are responsible for bone mineralization, endothelial cells form the inner part of blood vessels providing oxygen and nutrients to the emerging tissue^3^,^4^,^5^. Although those intelligent implants could be perfectly suited to control the growth of cells they might be less resistant to mechanical stress and thus inadequate for implants replacing joints. In this context, coatings of implant surfaces with amorphous, diamond-like carbon (DLC) received considerable attention in the past due to their superior mechanical properties^6^, superior biocompatibility towards mammalian cells^7^ and superior stability regarding wear^8^.

However, and unfortunately, also bacteria such as *Staphylococcus epidermidis* are able to colonize DLC surfaces^9^. *S. epidermidis* and also *S. aureus* are able to infect the surgical site during insertion of biomaterials associated with a high risk of life-threatening bacteraemia^10^. Therefore, the prophylactic treatment with systemic given antibiotics prior to the surgery is still recommended^11^. However, the formation of biofilms and resistant bacteria against antibiotics complicate the treatment of the affected patients dramatically^12^. This fact, forces the development of new materials for joint implants with innovative and effective antimicrobial strategies combined with high mechanical strength. In the past, several approaches have been followed to further improve the surface properties of DLC coatings in term of biocompatibility or to circumvent bacterial colonization. These include modifications with polytetrafluoroethylene (ptfe) providing bacterial repellent properties^9^,^13^, integration of titanium dioxide nanoparticles^14^ or doping with Silicon^15^. Hybrid surfaces containing silver were also shown to significantly reduce the adhesion of *S. epidermidis*^16^. Silver and silver nanoparticles are known to exert a high antimicrobial activity and have been employed for decades^17^,^18^,^19^. Nanocrystalline silver is currently investigated in several aspects ranging from wound healing^20^ to waste water treatment^21^. In contrast to resistances against antibiotics, microbes are largely lacking the potential to develop a silver resistance making it an ideal agent to control infection of bacterial strains such as *S. aureus*, prone to develop antibiotic resistances^22^. However, the broad antimicrobial effect of silver is potential harmful for the treated patient requiring the profound investigation of silver-mediated toxicity towards mammalian organisms^23^.

In the present study, we investigated novel intelligent hybrid implant surfaces composed of silver nanoparticles embedded within a DLC matrix. The aim of our study was the design of a surface characterized by a temporary release of reactive silver-ions (silver-burst) providing an antimicrobial activity and subsequently, after that silver burst, an excellent mammalian cell compatible surface.

## Results and Discussion

### Physical surface characterization

**Figure 1:**
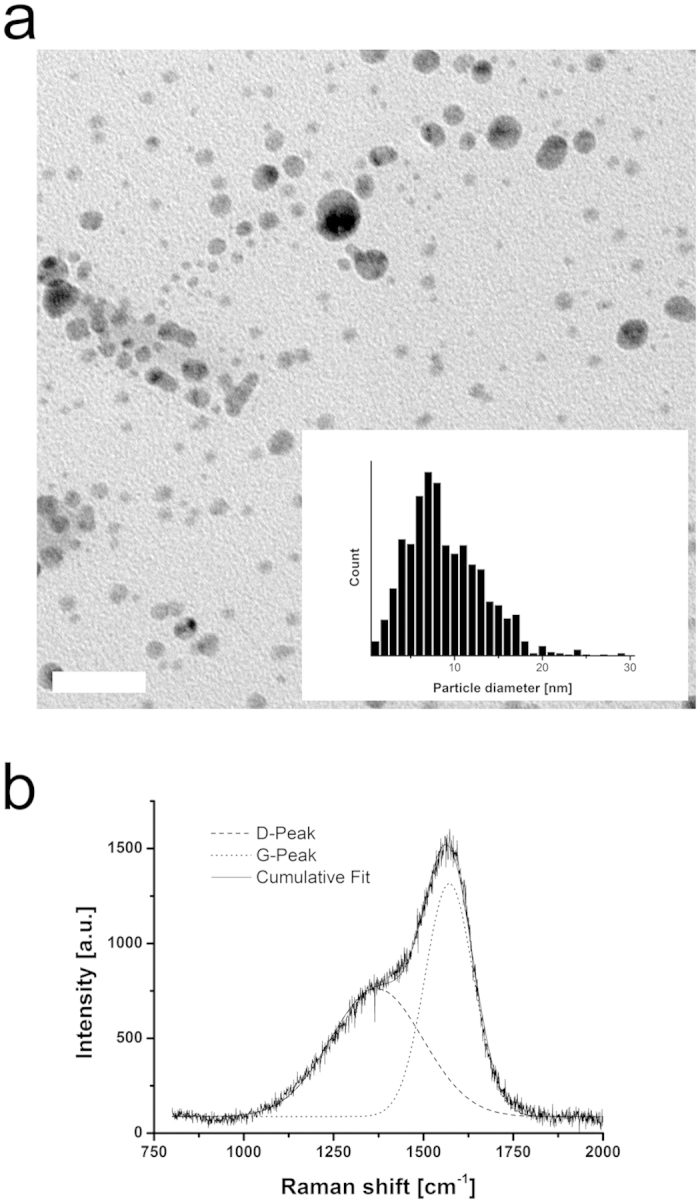
Physicochemical characterization of silver nanoparticles and DLC. (**a)** TEM image of silver particles in dispersion and particle size distribution determined from several images (inset). Scale bar corresponds to 50 nm. **(b)** Raman spectrum of PVP:Ag 1:2 sample, showing characteristic a-C:H signals. D stands for the breathing mode of six-fold rings (disorder) while G stands for the stretching mode of sp^2^-bonds.

Silver nanoparticle-containing DLC was produced on corundum-blasted medical grade Ti6Al4V by a three-step method including wet-chemical particle formation, dip-coating and polymer-to-DLC-transformation by plasma immersion ion implantation. Transmission electron micrograph (TEM) of a dispersion, containing silver nanoparticle is shown in Fig. 1a. The distribution of the particle diameter is given in the inset of Fig. 1a.

The thickness of the DLC coating was determined to be 60 ± 5 nm. Energy dispersive X-ray spectroscopy (EDX) mappings of the surface showed that the silver nanoparticles are present all over the surface, but with higher density on the sharp features of the rough titanium-blasted surface (Supplementary Fig. S1).

The chemical composition and density of the coatings are given in Table S1, as determined by Rutherford backscattering spectroscopy (RBS) and elastic recoil detection analysis (ERDA). As the table shows, layer density and hydrogen content are comparable to commonly deposited a-C:H^6^ and very similar in the different coatings, regardless of their silver content. The observed atomic percentages of silver in the coatings are also reflected by the initial molar ratios of silver nitrate (AgNO_3_) and polyvinylpyrrolidone (PVP) in the dispersions. Silver concentrations were defined by the ratio of PVP and AgNO_3_ during the preparation process. Highest achieved silver content within the DLC surface modification was 7.0 ± 0.5% (Ag:PVP ratio = 1:1; sample set A), lowest tested silver content was 0.9 ± 0.2% (Ag:PVP = 1:20; sample set D). Moreover, we investigated DLC surfaces with intermediate silver concentrations of 4.5 ± 0.5% (Ag:PVP = 1:2; sample set B) and 1.7 ± 0.4% (Ag:PVP = 1:10; sample set C). Due to its instability, not all experiments have been conducted on Ag:PVP 1:1 samples. Investigation of the bonding structure with Raman spectroscopy showed the typical D and G bonds of a-C:H (Fig. 1b). The intensity ratios of these two signals I(D)/I(G), obtained by fitting Gaussian functions, give estimated sp^3^-ratios of about 30%, which is also typical for a-C:H^24^,^25^. The nano hardness tests gave a hardness of 13.6 ± 3.8 GPa, which is a good value for a-C:H. Note that a very low indentation force of 1 mN had to be used in order to keep indentation depths under 10% of the total layer thickness, which leads to a relatively high margin of error here. An overview of all determined physical properties, which were independent of the silver concentration in the coatings is presented in Table S2, indicating the successful creation of a-C:H layers on Ti6Al4V substrates.

### Antimicrobial activity of implant surfaces

To investigate the antimicrobial properties of modified implant surfaces by silver nanoparticles integrated into a DLC layer, we selected two bacterial species, *Staphylococcus aureus* and *Staphylococcus epidermidis*. Both types of bacteria are prove to determine implant infections with a high prevalence^26^. The here tested strains were clinical isolates from previously affected patients, e.g. *S. epidermidis* (Catheter sepsis) and delivered from the ATCC biological resource centre and are thus appropriate to assess the antimicrobial properties of implant specimens^27^.

**Figure 2:**
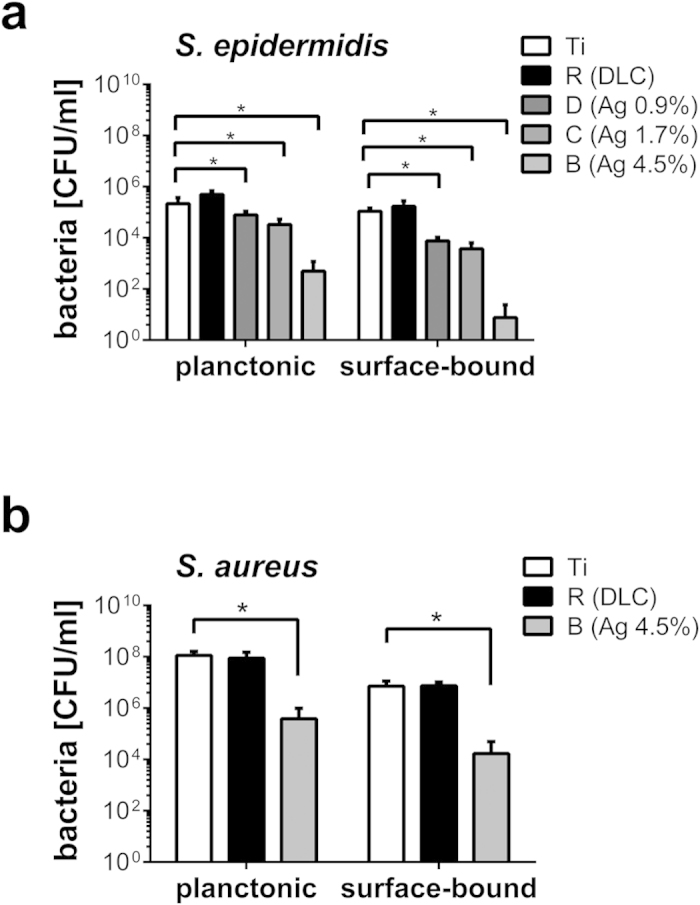
Antimicrobial activity of surface-modified implant specimens. Growth of planktonic and surface-bound *S. epidermidis* **(a)** and *S. aureus* **(b)** in the presence of indicated implant specimens. Concentration of bacteria is given as colony forming unit (CFU) and was measured 24 after inoculation with 10^5^ CFU/ml. Data are represented as mean ± SD of 16 independent experiments. Statistical significant difference (*P* < 0.05) is indicated by the asterisk. Ti = Ti6Al4V alloy; R = Ti6Al4V alloy with diamond-like carbon (DLC); B = DLC with 4.5 ± 0.5% silver; C = DLC with 1.7 ± 0.4% silver; D = DLC with 0.9 ± 0.2% silver.

To this end, we analyzed the growth of *S. epidermidis* and *S. aureus* on specimen surfaces (surface-bound) and in the supernatant (planktonic form in suspension). In comparison to silver-free specimens (sample set R) and DLC-coated surfaces, we found a significantly reduced growth of surface-bound *S. aureus* and *S. epidermidis* on specimens containing high silver concentrations at 4.5% (sample set B) 24 h after inoculation (Fig. 2a,b). In line with this finding we detected a reduced bacterial growth in the corresponding supernatant, suggesting a release of sufficient antibacterial amounts of silver into the bacterial suspension (Fig. 2a,b). We measured a dose-dependent relationship between the bactericidal properties of the specimens and the silver content. Growth of *S. epidermidis* was even reduced on surfaces containing only 0.9% silver (sample set D, Fig. 2a). Although *S. aureus* exhibited a higher level of silver tolerance in comparison to *S. epidermidis*, we could provide clear evidence that our silver nanoparticle-containing DLC coatings have antimicrobial properties. Silver susceptibility of microbes depends on species- or even strain-specific mechanisms reflecting variations in metabolizing silver or repairing silver-related cell damages. Antimicrobial properties of silver and silver nanocomposites towards many different microbes including also methicillin-resistant *S. aureus* (MRSA) have been well documented by others^28^,^29^,^30^,^31^. Interestingly, the acting molecular mechanisms of silver toxicity are complex and not limited to specific pathways offering therefore a broader antimicrobial profile and virtually no bacterial resistance in contrast to antibiotics^31^,^32^.

In previous studies we and others found a high cytotoxicity of silver and silver nanoparticles not only towards bacteria but also towards individual mammalian cells^32^,^33^,^34^ and the whole mammalian organism^23^. To further understand the cytotoxic properties of our implant modifications we next investigated growth of primary osteoblasts, endothelial cells and fibroblasts on silver-containing DLC.

### Silver-dependent cell growth on implant surfaces

We measured the toxicity of DLC surfaces with and without silver nanoparticles towards the mouse fibroblast-like cell line L929, primary human osteoblasts and primary human endothelial cells isolated form the umbilical cord vein (HUVEC).

**Figure 3:**
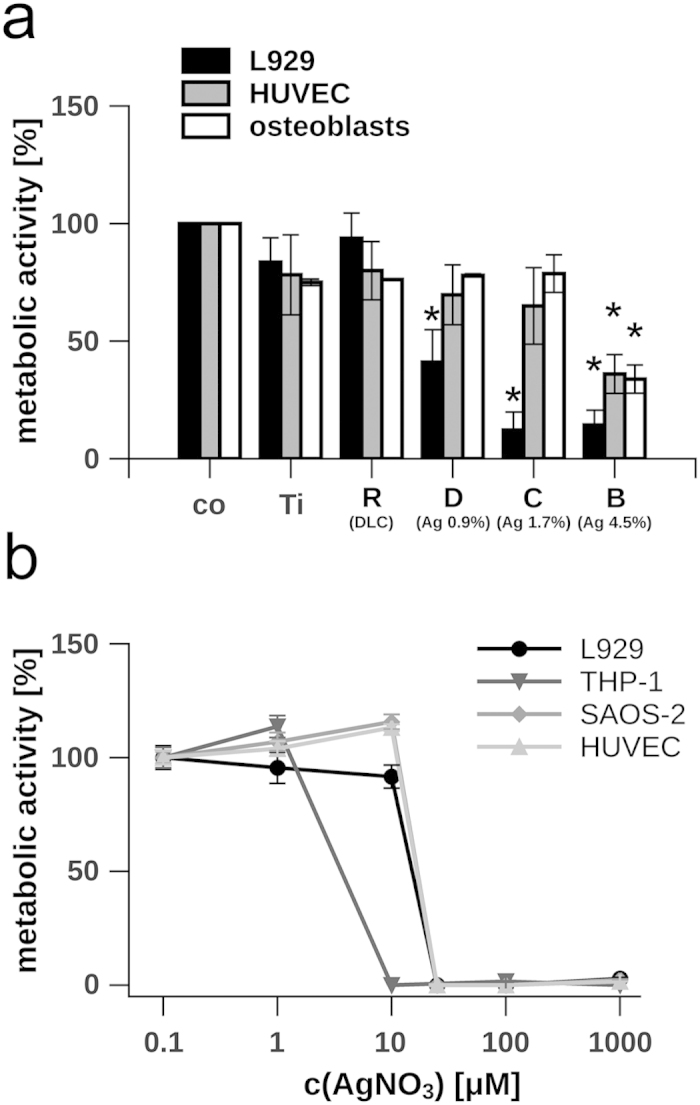
Evaluation of the metabolic activity of mammalian cells cultivated in the presence of silver. **(a)** L929 cells, primary osteoblasts and HUVEC were cultivated on different implants, while the metabolic activity was measured 24 h after seeding. The metabolic activity of cells grown on tissue culture-treated polystyrene (Co) was normalised to 100%, background levels of absorbance were subtracted. The silver content of the DLC is indicated in percentage. **(b)** Dose-dependent effect of silver nitrate (concentrations range from 0.1 to 1000 μM AgNO_3_) on the metabolism of L929, THP-1, SAOS-2 and HUVEC. Metabolic activity of non-treated cells was normalised to 100%, background levels of absorbance were subtracted. Data are represented as mean ± SD of at least 3 independent experiments. Statistical significant differences (*P* < 0.01) to the control group (co) are indicated by an asterisk. Ti = Ti6Al4V alloy; R = Ti6Al4V alloy with diamond-like carbon (DLC); B = DLC with 4.5 ± 0.5% silver; C = DLC with 1.7 ± 0.4% silver; D = DLC with 0.9 ± 0.2% silver.

Cellular viability was analyzed by WST-1 assay measuring the mitochondrial activity and thus metabolic activity of the cells (Fig. 3a). In preliminary experiments we verified the functionality of the WST-1 assay by comparative measurements of the intracellular ATP levels, of the cellular morphology and of the cellular DNA content (data not shown).

On native DLC coatings without incorporated silver (sample set R), we found that all tested cell types exhibit a high viability similar to cells cultivated on tissue-culture-treated polystyrene (co) (Fig. 3a). This finding is in accordance with former studies showing a high biocompatibility of DLC^35^. Growth inhibition of primary osteoblasts and HUVEC was only apparent on surfaces containing 4.5% silver (sample set B) while their metabolic activity was not significantly diminished on implant surfaces containing less silver. In contrast, L929 cells are more sensitive to silver since they grow already less on DLC containing only 0.9% silver (sample set D) (Fig. 3a).

Although we detected a comparable high resistance of HUVEC and osteoblasts towards silver-enriched DLC-coatings, our data clearly indicate that antimicrobial active coatings convey also a potentially unfavourable high toxicity toward mammalian cells^32^. To further distinguish the effect of silver ions and the DLC-surface properties on cell growth we analyzed the cellular metabolism in the presence of dissolved silver ions.

### Silver tolerance of mammalian cells

We measured the toxicity of silver nitrate towards the mouse fibroblast-like cell line L929, the human monocytic cell line THP-1, the osteoblast-like cell line SAOS-2 and HUVEC. THP-1 cells exhibit the lowest tolerance towards silver nitrate, since no measurable metabolism was apparent at concentrations of 10 μM. In contrast L929, SAOS-2 cells and HUVEC tolerate more than 10 μM AgNO3 (Fig. 3b). The experiments showed that except for the THP-1 cells, all other tested cell lines exhibit a very comparable silver tolerance.

Energy dispersive x-ray spectroscopy (EDX) mapping and raster electron microscopy (REM) (Supplementary Fig. S1) revealed a rough but homogeneous pattering of the surface regarding its chemical composition and its topography. Previous investigations of mammalian cell growth such as osteoblasts, endothelial or endothelial progenitor cells on scaffolds suggest a strong linkage between surface pattern^36^,^37^, surface properties^38^, and growth behaviour^39^ and might explain the striking difference between the cellular growth on silver nanoparticle enriched DLC-specimens (Fig. 3a) and cellular growth in the presence of dissolved silver ions (Fig. 3b).

### Growth behaviour on the implant surface

**Figure 4:**
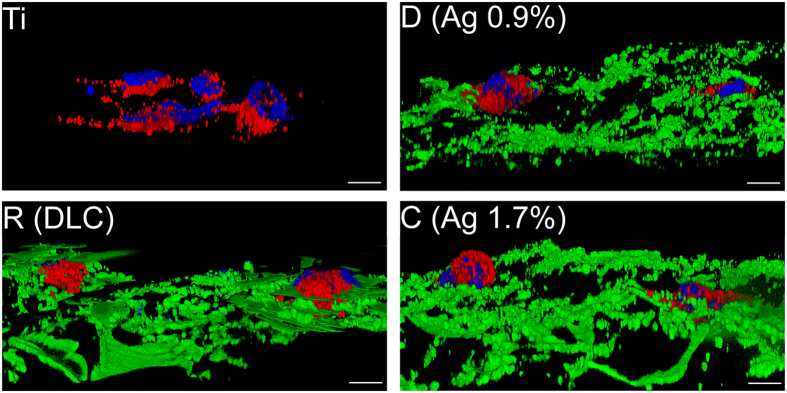
Analysis of endothelial cell growth on implant surfaces. Three-dimensional images of HUVEC grown on titanium (Ti), DLC (sample set R), DLC with 0.9% silver (sample set D) and DLC with 1.7% silver (sample set C). The endothelial cell marker von Willebrand factor (VWF) was stained in red. Nuclei were stained with DAPI (blue). DLC surfaces of implants are represented in green. White scale bars correspond to 10 μm.

Next, we applied scatter light-free structured illumination fluorescence microscopy (ApoTome), to investigate endothelial cell-specific morphology and cell integrity on DLC and DLC with silver in all three spatial dimensions. HUVEC were able to form cell clusters on the implant surface within the first 24 hour after seeding (Fig. 4) while a confluent cell layer developed within 7 days of cultivation (Supplementary Fig. S2). DLC-coated implants but not titanium surfaces are characterized by a strong green autofluorescence signal (525 ± 20 nm) upon excitation with light of a wavelength of 470 ± 20 nm (green structures, Fig. 4). To evaluate the differentiation status of the HUVEC we stained von Willebrand Factor (VWF) by immunofluorescence (red vesicles, Fig. 4). VWF plays a pivotal role in coagulation and inflammation and is therefore required for wound healing and tissue reconstruction^40^. VWF is stored in a ready releasable pool of large vesicles (Weibel-Palade-Bodies, WPB) within the endothelial cells and is co-released with a bulk of other factors comprising proangiogenic factors such as angiopoietin-2^41^. Therefore, the existence of VWF can be regarded as marker for endothelial cell functionality and its release as an initial event during wound healing crucial for the integration of implants into the bone tissue.

### Analysis of endothelial cell functionality

**Figure 5:**
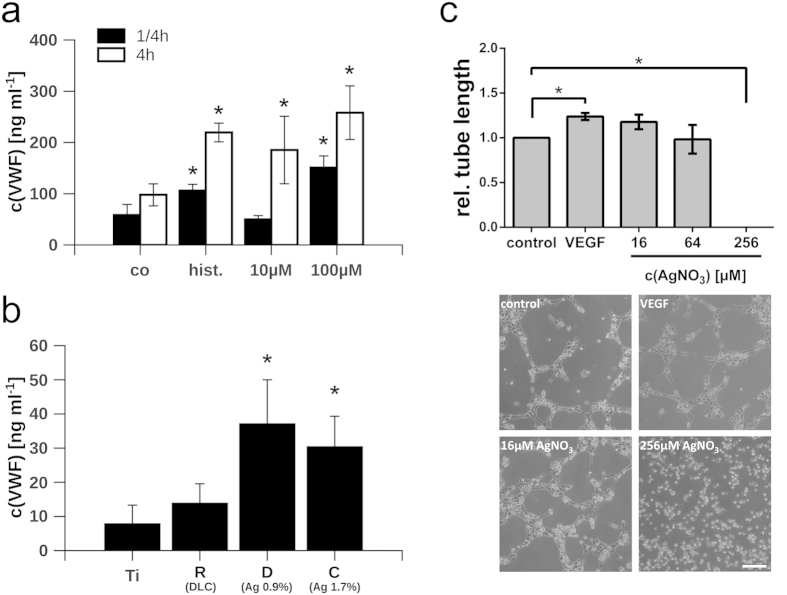
Analysis of VWF exocytosis. **(a)** Release of von Willebrand factor (VWF) after treatment with silver nitrate (10 μM and 100 μM) for 15 min (black bars) or 4 h (white bars). Co = tissue culture-treated polystyrene; hist. = 50μM histamine. **(b)** Release of VWF from HUVEC grown on implants. VWF release was induced by stimulation with 50μM histamine as positive control. The silver content of the DLC surfaces is indicated in percentage. **(c)** Relative average tube length of HUVEC in the presence of different silver nitrate concentrations (AgNO_3_) and VEGF-A (50 ng/ml) as a positive control. Representative images showing the tube formation capacity of HUVEC are depicted. Scale bar corresponds to 100 μm. Data are represented as mean ± SD of at least 3 independent experiments. Statistical significant differences (*P* < 0.01) to the control group (co) or titanium surfaces (Ti) are indicated by an asterisk. Ti = Ti6Al4V alloy; R = Ti6Al4V alloy with diamond-like carbon (DLC); B = DLC with 4.5 ± 0.5% silver; C = DLC with 1.7 ± 0.4% silver; D = DLC with 0.9 ± 0.2% silver.

To further understand the potency of endothelial cells for the regeneration of the injured bone tissue, we quantified the amount of endothelial-derived VWF secreted by HUVEC into the cell supernatant upon treatment with silver nitrate (Fig. 5a) or upon cultivation on implant surfaces (Fig. 5b). Stimulation with 50 μM histamine served as a positive control mediating a maximum of VWF exocytosis^42^. High concentrations of silver nitrate (100 μM) induced a strong release of VWF already 15 minutes after treatment. In contrast, we measured only a moderate increase of the VWF concentration in the supernatant after 4 h upon stimulation with low doses of silver (10 μM). In a previous study we have investigated the release of VWF from deceased or dying endothelial cells suggesting a correlation between the accumulation of VWF in the cellular supernatant and the reduction of the endothelial membrane integrity^43^. The fast and strong exudation of VWF upon high and toxic silver nitrate concentrations is thus likely related to the loss of the endothelial cell viability. However, the moderate release of VWF upon treatment with low concentrations of silver might point towards a controlled stimulation of HUVEC, a conclusion that is further underlined by the fact that at this low silver concentration also the proliferation was neither significantly increased nor decreased (Fig. 3b).

To measure the available amount of VWF stored within the cells cultivated on implants, we induced a maximal WPB exocytosis and thereby the acute release of its content through the treatment with histamine. As shown in Fig. 5b, a three-fold increase in the amount of VWF was detectable in the supernatants of cells cultivated on DLC-surface modifications with silver (sample sets C and D) when compared to pure titanium or DLC without silver (sample set R). These findings suggest an increased availability of VWF on DLC enriched with silver nanoparticles either due to an increased expression or a facilitated release.

To clarify whether silver affects the cell cycle and therefore the production of VWF, we correlated the DNA content per cell with the amount of VWF secreted into the cellular supernatant. Supplementary Fig. S3a,d indicate a linear connection between the release of VWF and the average cellular DNA content after treatment with silver nitrate or upon growth on the implants, suggesting a linkage between the cell cycle and the availability of VWF. In contrast, no such relationship was evident for the proinflammatory cytokine interleukin 6 (IL-6) (Supplementary Fig. S3b,c). Interestingly, nanocrystalline silver was found to have anti-inflammatory as well as pro-healing properties during skin regeneration after wounding or inflammatory diseases^44^,^45^ probably through the upregulation of growth factors^46^.

Our data provide clear evidence for a physiological growth, differentiation and functionality of endothelial cells cultivated on silver-containing implant coatings, while non-toxic concentrations of silver appears to even support a proangiogenic phenotype as indicated by an increased release of VWF. A tube formation assay could further prove this notion, since we found a clearly increased average tube length at low doses of silver nitrate (16μM) similar to that induced by recombinant vascular endothelial growth factor (VEGF). Only extremely high concentrations of silver (256 μM) could completely block the formation of tubes (Fig. 5c). Activation of endothelial cells and angiogenesis is fundamental for the proper wound healing and thus a key event during the bony integration of the implant. However, to allow colonization also of cells with a lower silver tolerance, a fast and complete release of silver from the DLC surface would be desired. To address this aspect, we analyzed the silver release kinetics of the DLC-silver implant surfaces in the next set of experiments.

### Silver release kinetics

Although endothelial cells resist comparable high silver concentrations (Fig. 3) proper integration of the implant into the bone tissue might be favoured in the absence of silver. To overcome these limited biocompatibility of silver containing DLC-coatings we proposed an intelligent surface providing a temporary silver burst suitable to prevent the colonization of the implant surface by bacteria peri-surgery and a fully mammalian cell compatible surface post-surgery. To verify the temporary release of silver from our DLC coatings we applied inductively coupled plasma optical emission spectroscopy (ICP-OES).

**Figure 6:**
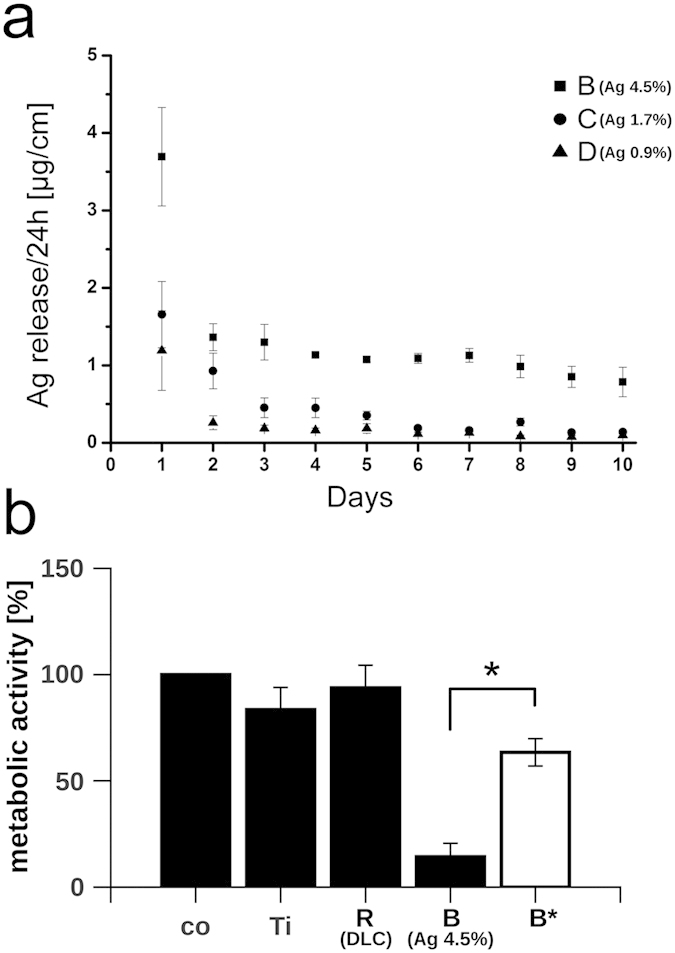
Silver release from silver nanoparticles embedded into DLC. **(a)** Silver release per area over ten days in simulated body fluid. **(b)** Metabolic activity of L929 cells cultivated on indicated specimens. The white bar reflects the metabolic activity of L929 cultivated on implants rinsed with water prior to the experiment. The metabolic activity of cells grown on tissue culture-treated polystyrene (co) was normalized to 100%, background levels of absorbance were subtracted. Data are represented as mean ± SD of at least 3 independent experiments. Statistical significant difference (*P* < 0.01) between the pretreated (B*) and the non-pretreated (B) specimens is indicated by the asterisk. Ti = Ti6Al4V alloy; R = Ti6Al4V alloy with diamond-like carbon (DLC); B = DLC with 4.5 ± 0.5% silver; C = DLC with 1.7 ± 0.4% silver; D = DLC with 0.9 ± 0.2% silver.

The analysis of the silver release kinetics into simulated body fluid showed highly time- and concentration-dependent release rates. Independent of the silver content available in the DLC surface, we measured a high initial silver release in the first 24 hours (Fig. 6a). In comparison to day 1, liberation of silver was significantly lower during day 2 and declined further in a linear manner in all tested sample sets. The amount of silver liberated from the specimens initially containing 0.9% (sample set D) and 1.7% (sample set C) silver, approaches the supposed lower detection limit of the used inductively coupled plasma optical emission spectroscopy (ICP-OES) of 0.14 μg/cm^2^ on day 7 and 9, respectively. The silver release of the DLC coating bearing the highest silver concentration (sample set B) decreases to 0.79 μg/cm^2^ on day 10, which is about 21% of the initial value. From a linear extrapolation of the release values of days 2 to 10, one can estimate that the release of this sample reaches the lower detection limit on day 20.

These results indicate that most of the available DLC-associated silver was released from the implant surface within the first hours after contact to body fluid. To further prove the biological consequence of the silver release, we simulated a peri-surgery release of silver from the DLC by rinsing the implant surface with sterile water prior to the dissemination of the silver-sensitive L929 cell. The results shown in Fig. 6b clearly demonstrate an almost non-affected growth of the cells on DLC nominally containing 4.5% silver (sample set B*) suggesting a biological relevant decay of cytotoxic silver after rinsing (metabolic activity on DLC (sample set R) = 94 ± 10.7%, metabolic activity on DLC with 4.5% silver (sample set B) = 14 ± 6.3% and metabolic activity on treated DLC with previously 4.5% silver (sample set B*) = 63 ± 6.4%) (Fig. 6b).

In summary, our results suggest an early and fast release of the silver from the implant surface and therefore a sufficient depression of bacterial vitality only peri-surgery. Additionally, our data indicate that the release of silver from the implant produces a biocompatible surface that appears to allow the early settlement of silver-sensitive cells already few hours post-surgery.

## Conclusion

The purpose of our work was the development of a novel implant coating having antimicrobial properties in combination with a high biological compatibility.

Therefore, we applied the previously developed plasma immersion ion implantation (PIII) of silver-polymer nanocomposites^47^ facilitating DLC modifications of three dimensional structures and envisioning also an industrial application. Moreover it is supposed that application of PIII improves the bond between the DLC and the implant surface reducing susceptibility to early delaminate due to a poor surface adhesion which is a major reason for implant revisions in clinics^48^.

Antimicrobial properties of DLC-silver composites have been investigated in previous works suggesting a useful approach to prevent colonization of bacteria^49^. In line with that, we could also demonstrate that our silver nanoparticle enriched DLC coatings exhibit antimicrobial activities due to the release of silver in a burst-like and transient manner, where the degree of toxicities was adjustable through the amount of incorporated silver nanoparticles.

**Figure 7:**
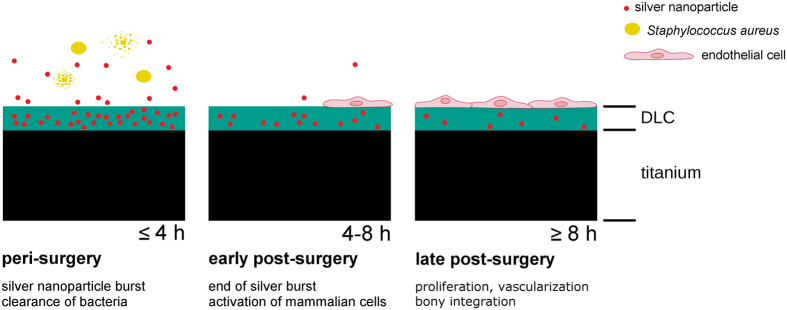
Schematic sketch of the major concepts of the intelligent implant surface. Early and excessive release of silver into the supernatant prevents the growth of bacteria such as *S. aureus* **(peri-surgery)**. Towards the end of the silver burst, low concentrations of silver nanoparticels induce a procoagulatory and a proangiogenic status in endothelial cells **(early post-surgery)**. Further cell proliferation and differentiation leads to vascularization and to the integration of the implant into the bone **(late post-surgery)**.

In addition we bought into focus the biological compatibility of DLC layers with and without incorporated silver in order to evaluate the potential of bony integration. In the past, various *in vitro* and *in vivo* studies could prove a high biocompatibility of DLC and its potential applicability in several clinical contexts including also orthopaedics^50^,^51^. Using *in vitro* settings, mostly growth of fibroblast and osteoblast and activation of monocytes/macrophages and platelets have been investigated^52^ while knowledge on endothelial cell growth and activation is still limited^53^. However, biocompatibility in terms of tissue vascularization is essential for the regeneration of the wounded bone after placement of a prosthesis as well as relevant for the proper bony integration of the implant post-surgery. Moreover, the potential impact of released silver ions on the tissue integration after surgery was largely unconsidered in the past prompting us to analyze the influence of silver on human endothelial cells regarding their potential to re- and neovascularize the tissue. Although we measured a clear cytotoxicity at high silver concentrations we measured that lower, nontoxic concentrations of silver could even promote a proangiogenic cell phenotype. Therefore, DLC with incorporated silver nanoparticles appears to be a convenient substrate that could prevent bacterial colonialization in a short-term and that could actively support tissue regeneration in the long-run. A schematic sketch summarizing the basic findings of our study is presented in Fig. 7.

## Methods

### Surface and material preparation and characterization

Surface and material preparation and characterization is outlined in detail in the supplementary material and elsewhere^54^.

### Measurement of silver release

Silver release kinetics were measured in phosphate buffered saline (PBS), simulating body fluid. The solution contained 8,000 mg/l NaCl, 200 mg/l KCl, 1,150 mg/l Na_2_HPO_4_ and 200 mg/l KH_2_PO_4_ (Dulbecco, Biochrom). For higher silver ion yield, three sample discs of the same silver concentration were put into a beaker glass at a time. In order to eliminate edge effects and contributions of the uncoated disc bottoms, the samples were embedded in paraffin. 10 ml PBS was added and incubated for 24 hours at 37 °C. Afterwards, the fluid was removed and replaced by another 10 ml of PBS. This was repeated for ten days and conducted with five sets of three discs for each concentration of silver in the coating. For comparison, one additional set of pure DLC without silver was used.

The incubated PBS solutions were analyzed by inductively coupled plasma optical emission spectroscopy (ICP-OES; Vista-MPX, Varian). A 10 μg/ml silver plasma standard solution (Alfa Aesar) was used for calibration.

### Antimicrobial activity

Antibacterial properties of the different surfaces were analyzed as previously described^27^,^32^. *Staphylococcus epidermidis* (ATCC^®^ 35984^TM^) and *S. aureus* (ATCC^®^ 25923^TM^) were cultured on Columbia Agar (CA) with 5% sheep blood (Becton-Dickinson, Heidelberg, Germany) at 37 °C overnight. After overnight culture on agar plates bacteria were diluted in Dulbeco’s Modified Eagle Medium (DMEM, life technologies, Paisley, GB) supplemented with 10% fetal calf serum and adjusted by densitometry with a McFarland 0.5 standard. Serial dilutions of the final suspension were plated on Columbia Agar with 5% sheep blood for a control of the inoculum counts. Surface modified specimens and untreated surfaces were then inoculated with 10^5^ CFU/ml (colony forming units) of bacteria in DMEM supplemented with 10% FCS for 24 h. Culture conditions were comparable for microbiologic and cell culture assays to guarantee the same concentrations of free metal ions in the growth medium. After the incubation interval, supernatant was removed and supplemented with a neutralizing solution to prevent reminiscent toxicity^24^. CFU were counted visually after 24 hours on CA plates to quantify antibacterial efficacy of released metal ions in the supernatant growth medium.

After careful rinsing of the colonized implant specimens to remove excessive bacteria, sonication was performed for 7 minutes (Sonorex RK255H, Bandelin Electronic, Berlin, Germany) in normal saline to remove the adhering microorganisms. Again, CFU were quantified on CA plates and after incubation for 24 hours. Complete detachment of the adhering microorganisms had been confirmed through scanning electron microscopy (SEM).

### Cell culture

L929 cells and THP-1 cells were maintained in RPMI 1640 medium supplemented with 10% fetal calf serum (FCS), 1% L-glutamine and 1% penicillin/streptomycin. SAOS-2 cells were grown in McCoy’s 5 medium with 10% FCS, 1% L-glutamine and 1% penicillin/streptomycin. Primary human osteoblast-like cells^25^ were cultured in calcium-free DMEM culture medium supplemented with 10% FCS, 2 mM glutamine, 1% penicillin/streptomycin and MEM-vitamins. Human umbilical vein endothelial cells (HUVEC) were freshly isolated from umbilical cord veins and cultivated in M199 medium supplemented with 10% FCS, and 1% growth supplement derived from bovine retina^55^. All cells were incubated at 37 °C in a humidified atmosphere containing 5% CO_2_.

### Mammalian cell activity after surface colonisation

Mitochondrial activity was measured with a WST-1 (1[2-(4-Iodophenyl)-3-(4-nitrophenyl)-5-(2,4-disulfophenyl)-2H-tetrazolium) assay kit (Roche, Mannheim, Germany). For the measurement of the intracellular concentrations of ATP, cells were harvested from the implant surface and were examined according to the manufacturer’s protocol (Promega, Mannheim, Germany).

### Scatter light-free structured illumination fluorescence microscopy (ApoTome)

Prior to ApoTome analyis VWF of HUVEC, grown on implant surfaces, was stained by immunofluorescence as previously described^43^. A detailed description is presented in the supplementary material.

### Quantification of von Willebrand factor

To quantify the concentration of von Willebrand factor (VWF) in the supernatant of HUVEC, we applied Enzyme Linked Immunosorbent Assays (ELISA) as previously reported^42^,^56^.

### Tube formation assay

Tube formation assays were conducted on matrigel plugs polymerized in angiogenesis cell culture dishes (ibidi, Martinsried, Germany). HUVECs were seeded in serum and growth factor free basal medium (EBM-2, Lonza, Cologne, Germany) at a cell concentration of 1 × 10^4^ per well. Where indicated, medium was supplemented with VEGF-A (50 ng/ml) or silver nitrate (16 μM, 64 μM, 256μM). Average length of tubes was measured after 4 h with a light microscope (Axiovert 100, Zeiss, Oberkochen, Germany).

### Statistical analysis

Values were expressed as the mean ± SD and correspond to at least three independent experiments. To test statistical significance the unpaired Student’s *t*-test was used. Results were considered as statistically different at *P* < 0.05.

## Additional Information

**How to cite this article**: Gorzelanny, C. *et al.* Silver nanoparticle-enriched diamond-like carbon implant modification as a mammalian cell compatible surface with antimicrobial properties. *Sci. Rep.* **6**, 22849; doi: 10.1038/srep22849 (2016).

## Supplementary Material

Supplementary Information

## References

[b1] Muller S. *et al.* VEGF-functionalized polyelectrolyte multilayers as proangiogenic prosthetic coatings. Adv Funct Mater 18, 1767–1775 (2008).

[b2] Zhang J.-T. *et al.* Stable Extracellular Matrix Protein Patterns Guide the Orientation of Osteoblast-like Cells. Adv Funct Mater 21, 4079–4087 (2011).

[b3] Mamalis A. A. & Cochran D. L. The therapeutic potential of oxygen tension manipulation via hypoxia inducible factors and mimicking agents in guided bone regeneration. A review. Archives of oral biology 56, 1466–1475 (2011). 21621191 10.1016/j.archoralbio.2011.05.001

[b4] Nguyen L. H. *et al.* Vascularized bone tissue engineering: approaches for potential improvement. Tissue engineering. Part B, Reviews 18, 363–382 (2012). 22765012 10.1089/ten.teb.2012.0012 PMC3458624

[b5] Sukul M., Nguyen T. B., Min Y. K., Lee S. Y. & Lee B. T. Effect of Local Sustainable Release of BMP2-VEGF from Nano-Cellulose Loaded in Sponge Biphasic Calcium Phosphate on Bone Regeneration. Tissue engineering. Part A 21, 1822–1836 (2015). 25808925 10.1089/ten.tea.2014.0497 PMC4449710

[b6] Robertson J. Diamond-like amorphous carbon. Materials Science and Engineering Reports 37, 129–281 (2002).

[b7] Thomson L. A., Law F. C., Rushton N. & Franks J. Biocompatibility of diamond-like carbon coating. Biomaterials 12, 37–40 (1991). 2009344 10.1016/0142-9612(91)90129-x

[b8] Thorwarth G. *et al.* Tribological behavior of DLC-coated articulating joint implants. Acta Biomaterialia 6, 2335–2341 (2010). 20018261 10.1016/j.actbio.2009.12.019

[b9] Kinnari T. J. *et al.* Adhesion of staphylococcal and Caco-2 cells on diamond-like carbon polymer hybrid coating. Journal of Biomedical Materials Research, Part A 86, 760–768 (2008). 18041722 10.1002/jbm.a.31643

[b10] AlBuhairan B., Hind D. & Hutchinson A. Antibiotic prophylaxis for wound infections in total joint arthroplasty: a systematic review. J Bone Joint Surg Br 90, 915–919 (2008). 18591602 10.1302/0301-620X.90B7.20498

[b11] Ericson C., Lidgren L. & Lindberg L. Cloxacillin in the prophylaxis of postoperative infections of the hip. J Bone Joint Surg Am 55, 808–813, 843 (1973). 4283753

[b12] Teterycz D. *et al.* Outcome of orthopedic implant infections due to different staphylococci. International Journal of Infectious Diseases 14, e913–e918 (2010). 20729115 10.1016/j.ijid.2010.05.014

[b13] Myllymaa K. *et al.* Formation and retention of staphylococcal biofilms on DLC and its hybrids compared to metals used as biomaterials. Colloids and Surfaces B: Biointerfaces 101, 290–297 (2013). 23010032 10.1016/j.colsurfb.2012.07.012

[b14] Marciano F. R. *et al.* Antibacterial activity of DLC films containing TiO2 nanoparticles. Journal of colloid and interface science 340, 87–92 (2009). 19758597 10.1016/j.jcis.2009.08.024

[b15] Liu C., Zhao Q., Liu Y., Wang S. & Abel E. W. Reduction of bacterial adhesion on modified DLC coatings. Colloids and Surfaces B: Biointerfaces 61, 182–187 (2008). 17897814 10.1016/j.colsurfb.2007.08.008

[b16] Katsikogianni M., Spiliopoulou I., Dowling D. P. & Missirlis Y. F. Adhesion of slime producing Staphylococcus epidermidis strains to PVC and diamond-like carbon/silver/fluorinated coatings. Journal of Materials Science: Materials in Medicine 17, 679–689 (2006). 16897160 10.1007/s10856-006-9678-8

[b17] Aziz Z., Abu S. F. & Chong N. J. A systematic review of silver-containing dressings and topical silver agents (used with dressings) for burn wounds. Burns 38, 307–318 (2012). 22030441 10.1016/j.burns.2011.09.020

[b18] Madhavan R. V., Rosemary M. J., Nandkumar M. A., Krishnan K. V. & Krishnan L. K. Silver nanoparticle impregnated poly (varepsilon-caprolactone) scaffolds: optimization of antimicrobial and noncytotoxic concentrations. Tissue engineering. Part A 17, 439–449 (2011). 20807004 10.1089/ten.TEA.2009.0791

[b19] Berean K. J. *et al.* A unique *in vivo* approach for investigating antimicrobial materials utilizing fistulated animals. Scientific reports 5, 11515 (2015). 26098413 10.1038/srep11515 PMC4476420

[b20] Riehemann K. *et al.* Nanomedicine-Challenge and Perspectives. Angewandte Chemie International Edition 48, 872–897 (2009). 10.1002/anie.200802585 PMC4175737 19142939

[b21] Swathy J. R. *et al.* Antimicrobial silver: an unprecedented anion effect. Scientific reports 4, 7161 (2014). 25418185 10.1038/srep07161 PMC4241523

[b22] Franci G. *et al.* Silver Nanoparticles as Potential Antibacterial Agents. Molecules 20, 8856–8874 (2015). 25993417 10.3390/molecules20058856 PMC6272636

[b23] Yoisungnern T. *et al.* Internalization of silver nanoparticles into mouse spermatozoa results in poor fertilization and compromised embryo development. Scientific reports 5, 11170 (2015). 26054035 10.1038/srep11170 PMC4459204

[b24] Tilton R. C. & Rosenberg B. Reversal of the silver inhibition of microorganisms by agar. Applied and Environmental Microbiology 35, 1116–1120 (1978). 354526 10.1128/aem.35.6.1116-1120.1978 PMC242993

[b25] Magdolen U. *et al.* Growth promoting *in vitro* effect of synthetic cyclic RGD-peptides on human osteoblast-like cells attached to cancellous bone. International journal of molecular medicine 17, 1017–1021 (2006). 16685410

[b26] Trampuz A. & Zimmerli W. Diagnosis and treatment of infections associated with fracture-fixation devices. Injury 37, 59–66 (2006). 10.1016/j.injury.2006.04.010 16651073

[b27] Harrasser N. *et al.* Antibacterial efficacy of ultrahigh molecular weight polyethylene with silver containing diamond-like surface layers. AMB Express 5, 64 (2015). 26391393 10.1186/s13568-015-0148-x PMC4577490

[b28] Bera R. K., Mandal S. M. & Raj C. R. Antimicrobial activity of fluorescent Ag nanoparticles. Letters in applied microbiology 58, 520–526 (2014). 24460988 10.1111/lam.12222

[b29] Matai I. *et al.* Antibacterial activity and mechanism of Ag-ZnO nanocomposite on S. aureus and GFP-expressing antibiotic resistant *E. coli*. Colloids and surfaces. B, Biointerfaces 115, 359–367 (2014). 24412348 10.1016/j.colsurfb.2013.12.005

[b30] Khurana C., Vala A. K., Andhariya N., Pandey O. P. & Chudasama B. Antibacterial activity of silver: the role of hydrodynamic particle size at nanoscale. Journal of biomedical materials research. Part A 102, 3361–3368 (2014). 24166739 10.1002/jbm.a.35005

[b31] de Moraes A. C., Lima B. A., de Faria A. F., Brocchi M. & Alves O. L. Graphene oxide-silver nanocomposite as a promising biocidal agent against methicillin-resistant Staphylococcus aureus. International journal of nanomedicine 10, 6847–6861 (2015). 26586946 10.2147/IJN.S90660 PMC4636171

[b32] Heidenau F. *et al.* A novel antibacterial titania coating: Metal ion toxicity and *in vitro* surface colonization. Journal of Materials Science: Materials in Medicine 16, 883–888 (2005). 16167096 10.1007/s10856-005-4422-3

[b33] Smoot E. C. 3rd, Kucan J. O., Roth A., Mody N. & Debs N. *In vitro* toxicity testing for antibacterials against human keratinocytes. Plast Reconstr Surg 87, 917–924 (1991). 2017501 10.1097/00006534-199105000-00017

[b34] Steffensen I. L. *et al.* Cytotoxicity and accumulation of Hg, Ag, Cd, Cu, Pb and Zn in human peripheral T and B lymphocytes and monocytes *in vitro*. Gen Pharmacol 25, 1621–1633 (1994). 7721038 10.1016/0306-3623(94)90364-6

[b35] Amaral M. *et al.* Cytotoxicity evaluation of nanocrystalline diamond coatings by fibroblast cell cultures. Acta Biomaterialia 5, 755–763 (2009). 18819854 10.1016/j.actbio.2008.08.015

[b36] Lu J., Rao M. P., MacDonald N. C., Khang D. & Webster T. J. Improved endothelial cell adhesion and proliferation on patterned titanium surfaces with rationally designed, micrometer to nanometer features. Acta Biomaterialia 4, 192–201 (2008). 17851147 10.1016/j.actbio.2007.07.008

[b37] Zhu B., Lu Q., Yin J., Hu J. & Wang Z. Alignment of osteoblast-like cells and cell-produced collagen matrix induced by nanogrooves. Tissue engineering 11, 825–834 (2005). 15998222 10.1089/ten.2005.11.825

[b38] An N. *et al.* Proliferation, behavior, and cytokine gene expression of human umbilical vascular endothelial cells in response to different titanium surfaces. Journal of Biomedical Materials Research, Part A 93, 364–372 (2010). 19569217 10.1002/jbm.a.32539

[b39] Ziebart T. *et al.* Interactions between endothelial progenitor cells (EPC) and titanium implant surfaces. Clinical Oral Investigations 17, 301–309 (2012). 22406922 10.1007/s00784-012-0691-7

[b40] Petri B. *et al.* von Willebrand factor promotes leukocyte extravasation. Blood 116, 4712–4719 (2010). 20716766 10.1182/blood-2010-03-276311

[b41] Fiedler U. *et al.* The Tie-2 ligand angiopoietin-2 is stored in and rapidly released upon stimulation from endothelial cell Weibel-Palade bodies. Blood 103, 4150–4156 (2004). 14976056 10.1182/blood-2003-10-3685

[b42] Goerge T. *et al.* Tumor-derived matrix metalloproteinase-1 targets endothelial proteinase-activated receptor 1 promoting endothelial cell activation. Cancer research 66, 7766–7774 (2006). 16885380 10.1158/0008-5472.CAN-05-3897

[b43] Bauer A. T. *et al.* Cytotoxicity of silica nanoparticles through exocytosis of von Willebrand factor and necrotic cell death in primary human endothelial cells. Biomaterials 32, 8385–8393 (2011). 21840590 10.1016/j.biomaterials.2011.07.078

[b44] Wright J. B., Lam K., Buret A. G., Olson M. E. & Burrell R. E. Early healing events in a porcine model of contaminated wounds: effects of nanocrystalline silver on matrix metalloproteinases, cell apoptosis, and healing. Wound repair and regeneration: official publication of the Wound Healing Society [and] the European Tissue Repair Society 10, 141–151 (2002). 10.1046/j.1524-475x.2002.10308.x 12100375

[b45] Demling R. H. & Leslie DeSanti M. D. The rate of re-epithelialization across meshed skin grafts is increased with exposure to silver. Burns 28, 264–266 (2002). 11996859 10.1016/s0305-4179(01)00119-x

[b46] Nadworny P. L., Wang J., Tredget E. E. & Burrell R. E. Anti-inflammatory activity of nanocrystalline silver-derived solutions in porcine contact dermatitis. Journal of inflammation 7, 13 (2010). 20170497 10.1186/1476-9255-7-13 PMC2841158

[b47] Schwarz F. & Stritzker B. Plasma immersion ion implantation of polymers and silver-polymer nano composites. Surf Coat Tech 204, 1875–1879 (2010).

[b48] Hauert R., Thorwarth K. & Thorwarth G. An overview on diamond-like carbon coatings in medical applications. Surf Coat Tech 233, 119–130 (2013).

[b49] Love C. A., Cook R. B., Harvey T. J., Dearnley P. A. & Wood R. J. K. Diamond like carbon coatings for potential application in biological implants-a review. Tribol. Int. 63, 141–150 (2013).

[b50] Dearnaley G. & Arps J. H. Biomedical applications of diamond-like carbon (DLC) coatings: A review. Surf Coat Tech 200, 2518–2524 (2005).

[b51] Brennan S. A. *et al.* Silver nanoparticles and their orthopaedic applications. The bone & joint journal 97-B, 582–589 (2015). 25922449 10.1302/0301-620X.97B5.33336

[b52] Roy R. K. & Lee K. R. Biomedical applications of diamond-like carbon coatings: A review. J. Biomed. Mater. Res. Part B 83B, 72–84 (2007). 10.1002/jbm.b.30768 17285609

[b53] Maguire P. D. *et al.* Mechanical stability, corrosion performance and bioresponse of amorphous diamond-like carbon for medical stents and guidewires. Diam. Relat. Mat. 14, 1277–1288 (2005).

[b54] Schwarz F. & Stritzker B. Plasma immersion ion implantation of polymers and silver–polymer nano composites: Proceedings of the European Materials Research Socierty (E-MRS)Spring Meeting 2009 Symposium P. Surface and Coatings Technology 204, 1875–1879 (2010).

[b55] Goerge T. *et al.* Secretion pores in human endothelial cells during acute hypoxia. The Journal of Membrane Biology 187, 203–211 (2002). 12163978 10.1007/s00232-001-0164-4

[b56] Gorzelanny C., Pöppelmann B., Pappelbaum K., Moerschbacher B. M. & Schneider S. W. Human macrophage activation triggered by chitotriosidase-mediated chitin and chitosan degradation. Biomaterials 31, 8556–8563 (2010). 20797781 10.1016/j.biomaterials.2010.07.100

