# Supplementary material for: Silver nanoparticle-enriched diamond-like carbon implant modification as a mammalian cell compatible surface with antimicrobial properties
Source: Sci Rep. 2016 Mar 9;6:22849. doi: 10.1038/srep22849 (PMC4783782; doi:10.1038/srep22849)
Supplement: Supplementary Information [file srep22849-s1.pdf]

Supplementary information

for

Silver nanoparticle-enriched diamond-like carbon implant modification as a mammalian cell compatible surface with antimicrobial properties

Christian Gorzelanny[a], Ralf Kmeth[b], Andreas Obermeier[c], Alexander T. Bauer[a], Natalia Halter[a], Katharina Kümpel[c], Matthias F. Schneider[d], Achim Wixforth[e,f], Hans Gollwitzer[c], Rainer Burgkart[c], Bernd Stritzker[b], and Stefan W. Schneider[a,]*

[a] Experimental Dermatology, Department of Dermatology, Medical Faculty Mannheim, Heidelberg University, Mannheim, Germany

[b] Experimental Physics IV, Physics Institute, Augsburg University, Augsburg, Germany

[c] Clinic of Orthopedics and Traumatology, Technical University Munich, 81675 Munich, Germany

[d] Department of Mechanical Engineering, Boston University, Boston, MA 02215, US

[e] Experimental Physics I, Physics Institute, Augsburg University, Augsburg, Germany

[f] Augsburg Center for Innovative Technologies, Augsburg, Germany

***: corresponding author:**

Prof. Stefan W. Schneider, Experimental Dermatology, Department of Dermatology, Medical Faculty Mannheim, Heidelberg University, 68167 Mannheim (Germany)

E-mail: stefan.schneider@medma.uni-heidelberg.de

Methods

Surface preparation and characterization

For the purpose of preparing silver nanoparticle-containing DLC coatings, a hybrid synthesis method based on the ion-induced transformation of silver–polymer nanocomposites has been used [1].

The preparation of the silver-containing DLC-coatings is basically divided into three steps which are described in detail below. Five sample sets were coated differently, including four concentrations of silver in the coating (sample sets A, B, C and D) and one silver-free DLC-coating (sample set R). In addition, a set of uncoated substrates was used as a reference. As substrate, corundum-blasted medical Ti6Al4V discs (AST; F 136, MedTitan, R_a~5µm) with 10 mm diameter and 2 mm thickness were used in order to simulate bone implant surfaces. For material characterisation, non-blasted discs were used.

Colloidal Ag nanoparticle dispersion

As a first step, a colloidal dispersion of stabilized silver nanoparticles has been synthesised. In this step, Ag concentration and nanoparticle sizes in the final coating can be controlled through the concentration of the educts. Silver nanoparticles were synthesized by photochemical reduction of silver nitrate ($AgNO_3$) dissolved in a mixture of ethanol and acetone. The addition of benzoin, which splits into a benzyl radical and a benzoin radical under UV irradiation, leads to a more homogenous particle distribution compared to a reduction in an alcoholic solution [2,3]. The particles were stabilized by steric hindrance, using polyvinylpyrrolidone (PVP). As large sample sets were needed for biological characterization of the samples (each set consisted of 150 samples), long-time stable dispersions had to be prepared. Dispersions described in earlier publications [4] turned out not to be long-time stable, as their silver concentration started decreasing after only one day due to precipitation of silver particles. Lower molar ratios of benzoin to $AgNO_3$ (benzoin:$AgNO_3$ 1:2) as well as constant

concentrations of the basic solutions ($AgNO_3$ in ethanol 0,1 mol/l; PVP in ethanol 0,6 mol/l; benzoin in aceton 0,05 mol/l) proved to give better stability, with constant silver concentrations for more than five days. Particle sizes, however, were bigger when compared to dispersions of higher molar ratio, independent of the used silver concentration. Fig. 1 shows a TEM bright field image of the silver particles, which were observed on a polyvinylformal-coated copper grid that was dip-coated in the dispersion, with an average particle diameter of 9 nm and a maximum diameter of 30 nm. Four different silver concentrations were prepared (Ag:PVP molar ratio in the solution 1:1, 1:2, 1:10 and 1:20), along with one pure PVP solution without silver. The solutions were stirred and de-gassed in an ultrasonic bath under exclusion of light and afterwards exposed to UV light (Hg vapor lamp) for 6 hours under N_2 atmosphere to initiate the photochemical reduction. As the 1:1 solutions showed precipitations with inconstant and lower than the expected silver concentrations, they were not used throughout the whole study.

Dip-coating

PVP polymer films with the different Ag concentrations were deposited by dip-coating. Film thickness was controlled by retraction speed, resulting in a highly homogenous coating with significant deviations only at the sample edges. All sample sets were coated with a polymer film of 130 nm thickness.

Plasma immersion ion implantation

An experimental vacuum set-up equipped with a magnetron microwave generator (Muegge), an ECR magnet and a high voltage pulse generator (GBS Elektronik) was used for PIII. Earlier experiments showed optimal polymer-to-DLC transformation parameters for Ne implantation with 20keV implantation energy and a fluence of $5 \cdot 10^{16}$ ions/cm^2. Ne and CH_4 were used as processing gases with a ratio of 2.5:1. In this way, the hydrocarbon species

deposited out of the plasma provide compensation for sputtering losses. Note that this ratio is highly equipment specific, as in another PIII set-up it was 1.5:1. The working gas pressure was adjusted to 0.5 Pa. 800W power were applied to the microwave generator and 6A current to the ECR magnet to generate the plasma. High voltage pulses of -20kV with 5 µs duration and 200 Hz repetition rate were applied in order to conduct PIII.

Material characterization

Sample characterization included film thickness measurement by profilometry, nano hardness testing, Rutherford backscattering spectroscopy (RBS), Raman spectroscopy and electron microscopy (SEM and TEM). As most characterization techniques cannot be conducted on samples with a high surface roughness, polished Ti6Al4V substrates had to be used instead of the corundum-blasted specimens. Sample composition and areal density were determined by RBS and ERDA, utilizing 1.8 MeV He^+ ions at an angle of incidence of 70° with a Tandetron accelerator. Mass density was calculated from film thickness and areal atomic density, taking into account only H and C atomic fractions, as the contribution of O and N can be neglected. In order to obtain pure carbon matrix densities, Ag fractions were disregarded as well in case of silver-containing samples.

Micro Raman spectroscopy was used to investigate the carbon bonding structure of the samples. The applied coatings were excited with the 488 nm line of an argon ion laser of 2 mW beam power. The illumination spot size was about 2 µm, focusing through a 50x microscope objective. The spectra were recorded using a CCD based spectrometer (T64000, Instruments S.A.).

Nano hardness testing of the coatings was conducted on a nanoindentation system (UNAT, Asmec). Indention force was 1 mN, applied with a Berkovich tip.

The distribution of the silver particles over the surface was observed via energy dispersive x-ray spectroscopy (EDX) on a SEM (ESEM XL 30 FEG, FEI).

The morphology of the nanoparticles in the film was analyzed using cross-sectional TEM. The images were taken on a 200 keV high-resolution field emission TEM (JEM2100F, JEOL) equipped with an imaging filter (Gatan).

Scatter light-free structured illumination fluorescence microscopy (ApoTome)

HUVEC, grown on implant surfaces, were fixed with ice-cold methanol for 30 min, washed with HEPES-buffered Ringer solution (140 mM NaCl, 5 mM KCl, 1 mM $MgCl_2$; 1 mM $CaCl_2$, 5 mM glucose and 10 mM HEPES) and blocked with 2% bovine serum albumin (BSA) dissolved in HEPES-buffered Ringer solution, supplemented with 0.3% Triton x-100 for 1 h at room temperature. Incubation with the primary antibody rabbit anti-human VWF (DakoCytomation, Glostrup, Denmark) was performed with a dilution of 1:200 in incubation buffer (HEPES-buffered Ringer solution supplemented with 0.1% BSA) for 1 h at room temperature. After washing, cells were incubated with a secondary Qdot-conjugated goat anti-rabbit IgG antibodies (Life Technologies GmbH, Darmstadt, Germany) diluted 1:400 in incubation buffer at room temperature for 1 h. Nuclei were stained for 10 min with 4,6-diamidino-2-phenylindole (DAPI) diluted in incubation buffer (0.1 µg ml^{-1}). Finally, implants were embedded with mowiol–glycol solution with freshly added 50 mg/ml DABCO (1.4-diazabicyclo-[2.2.2] octane; Sigma Aldrich, St. Louis, US). Three-dimensional microscopy was employed using a Zeiss Z.1 observer equipped with an ApoTome module (Zeiss, Jena, Germany) under a 20x or 40x oil-immersion objective.

References

1 Schwarz, F. & Stritzker, B. Plasma immersion ion implantation of polymers and silver–polymer nano composites: Proceedings of the European Materials Research Socierty (E-MRS)Spring Meeting 2009 Symposium P. *Surface and Coatings Technology* **204**, 1875–1879, (2010).

2 Ayyappan, S., Gopalan, R. S., Subbanna, G. N. & Rao, C. N. R. Nanoparticles of Ag, Au, Pd, and Cu produced by alcohol reduction of the salts. *Journal of Materials Research* **12**, 398-401, (1997).

3 Itakura, T., Torigoe, K. & Esumi, K. Preparation and Characterization of Ultrafine Metal Particles in Ethanol by UV Irradiation Using a Photoinitiator. *Langmuir* **11**, 4129-4134, (1995).

4 Schwarz, F., Thorwarth, G. & Stritzker, B. Synthesis of silver and copper nanoparticle containing a-C:Hby ion irradiation of polymers. *Solid State Sciences* **11**, 1819-1823, (2009).

Table S1: Chemical composition of the prepared layers

Sample set	Ag:PVP ratio in solution	Ag concentration in layer [at%]	Layer matrix density [g/cm³]	Chemical composition of layer matrix [at%]			
				C	O	N	H
A	1:1	7.0 ± 0.5	1.9 ± 0.3	53.9	5.2	5.4	35.5
B	1:2	4.5 ±0.5	1.6 ± 0.2	56.2	4.9	5.2	33.7
C	1:10	1.7 ±0.4	1.7 ± 0.2	55.1	5.4	5.1	34.4
D	1:20	0.9 ± 0.2	1.7 ± 0.1	55.7	5.2	4.8	34.2
R	-	-	1.7 ± 0.2	56.0	4.8	5.1	34.1

Table S2: Physical details of the applied DLC layers

Thickness of DLC layer [nm]	Layer density [g·cm^{-3}]	Hydrogen content [at%]	sp^3 bonding ratio	Nanohardness [GPa]
60 ± 5	1.7 ± 0.2	34 ± 3	0.35 ± 0.05	14 ± 2

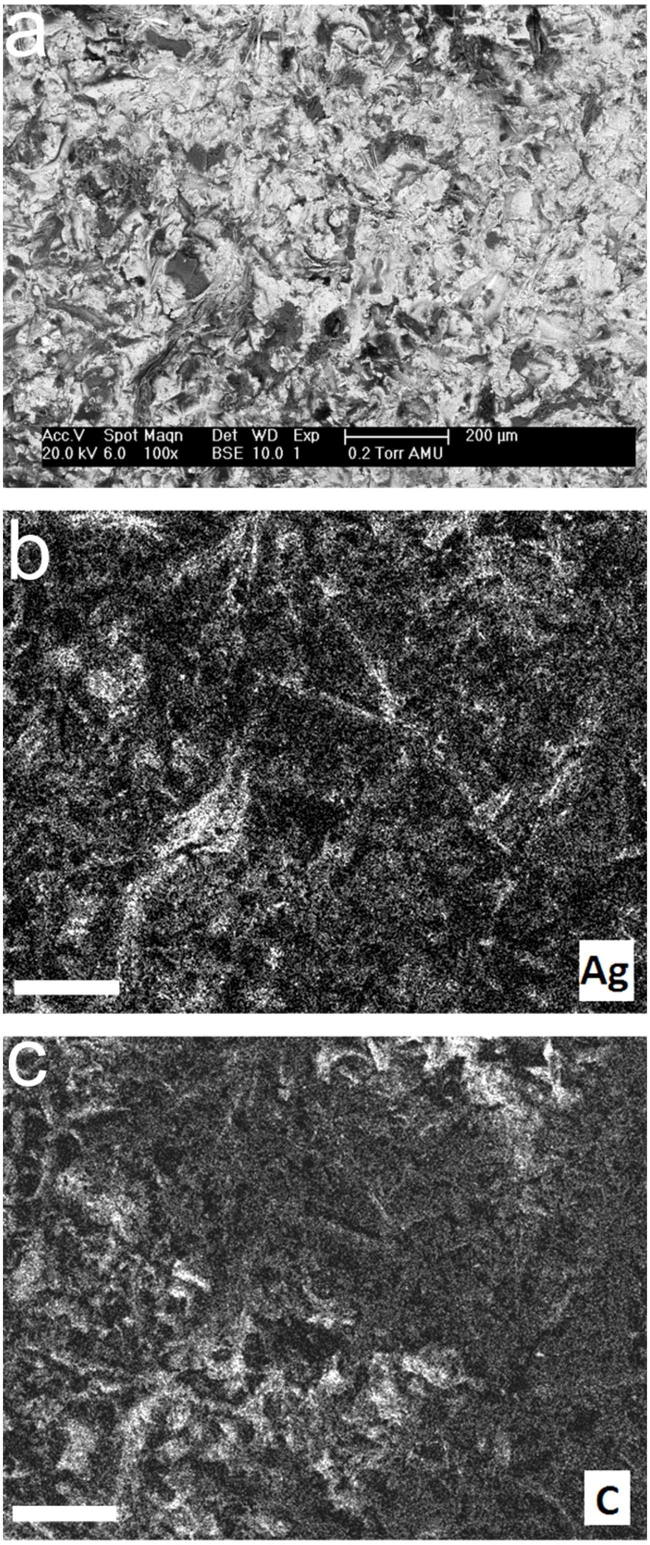

Fig. S1. Topography and chemical composition of the DLC layer. (a) SEM image of the corundum-blasted Ti6Al4V surface and **(b-c)** EDX mapping images of silver (Ag) and carbon (C) on the same position

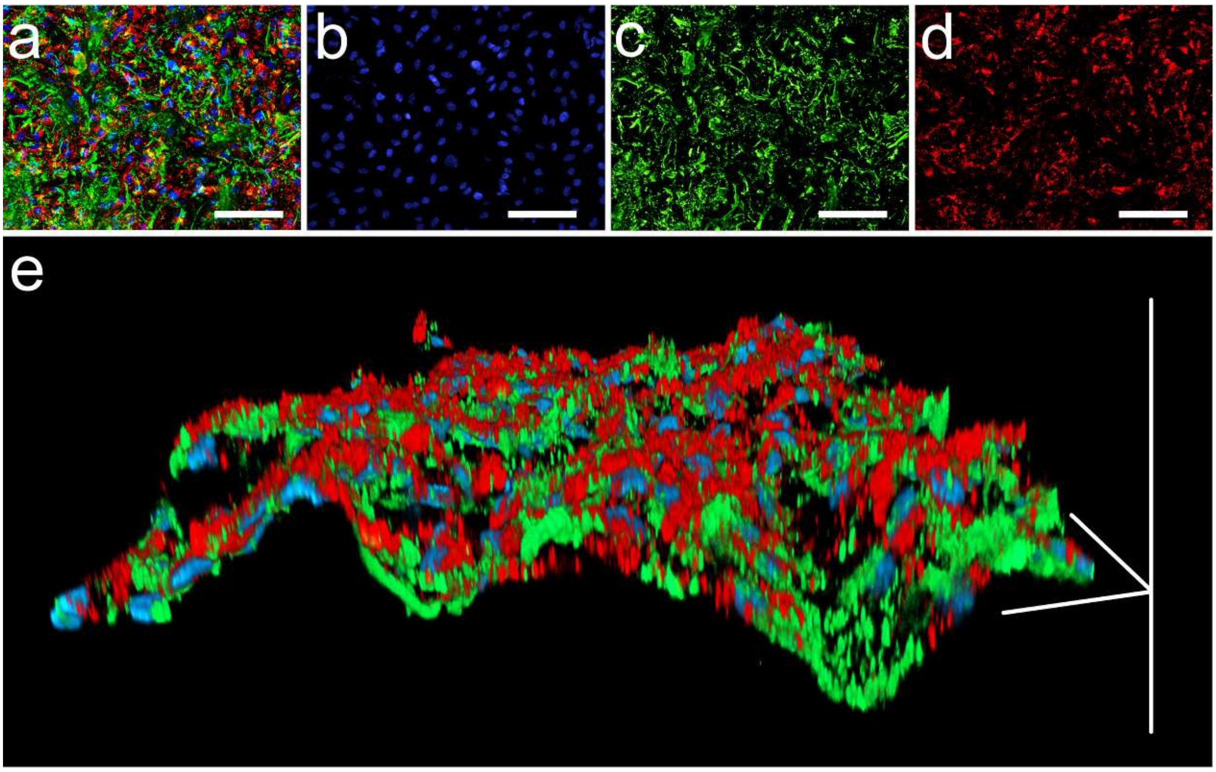

Fig. S2. HUVEC form a confluent cell layer on DLC containing 4.5% silver (sample set B). (a) Merged two-dimensional fluorescence image of the implant surface, **(b-d)** represent single fluorescence channels superimposed in the merged image. The blue color **(b)** shows the nuclear staining with DAPI, the green color **(c)** corresponds to the green autofluorescence signal generated by the DLC and the red color **(d)** reflects the intercellular staining of VWF. Scale bars correspond to 100 µm. **(e)** Three-dimensional fluorescence image of the implant surface shown in **(a)**. Vertical scale bars correspond to 60 µm. Horizontal scale bar corresponds to 65 µm.

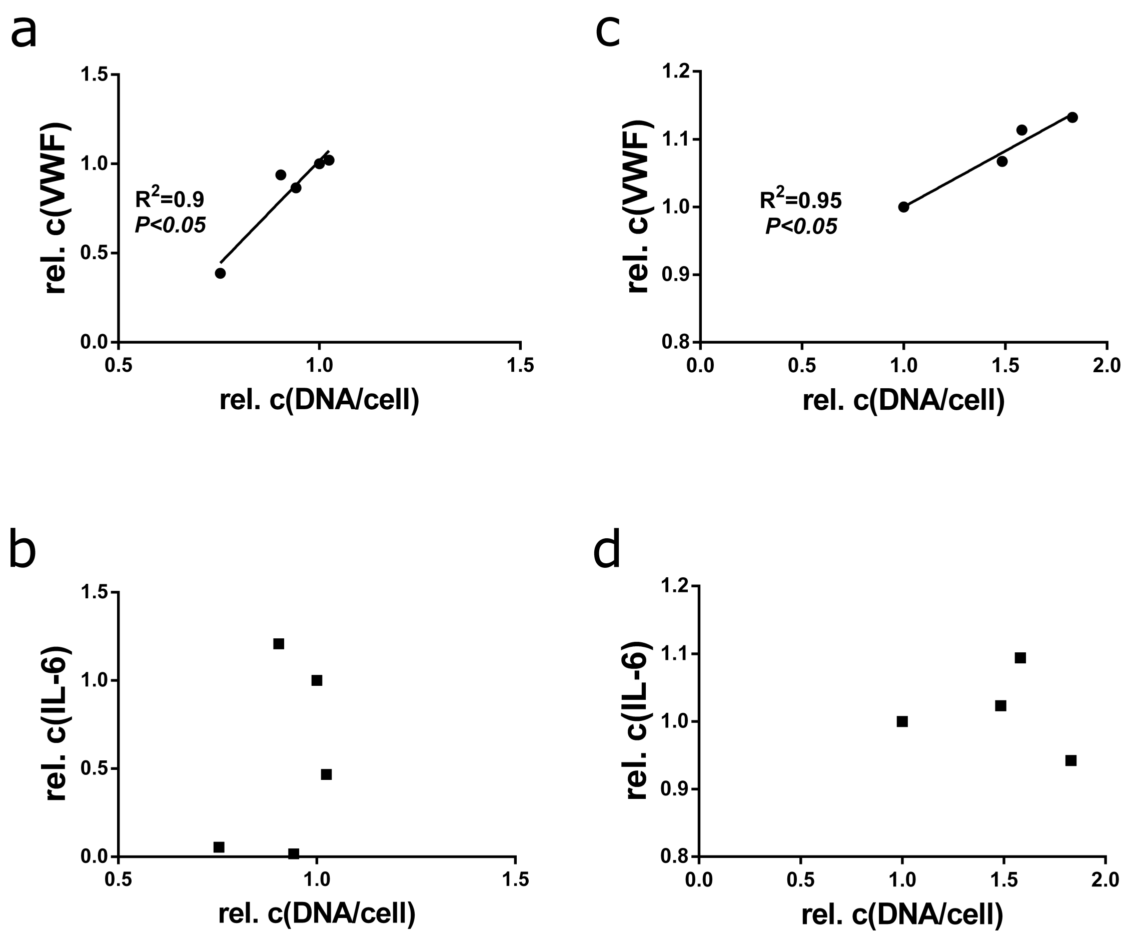

Fig. S3. Correlation between the cellular DNA content of HUVEC and the release of VWF and IL-6. (a) Amount of released VWF from HUVEC was measured upon treatment with increasing concentrations of $AgNO_3$ (4 µM, 16 µM 64 µM and 256 µM) as a function of the average DNA content per cell. DNA content was quantified through fluorescence microscopic analyses of DAPI-stained cell nuclei. **(b)** Amount of released IL-6 from HUVEC was measured upon treatment with increasing concentrations of $AgNO_3$ (4 µM, 16 µM 64 µM and 256 µM) as a function of the average DNA content per cell. **(c)** Amount of released VWF from HUVEC cultivated on different implant subsets (sample set: Ti, R, D and C) as a function of the average DNA content per cell. **(d)** Amount of released IL-6 from HUVEC cultivated on different implant subsets (sample set: Ti, R, D and C) as a function of the average DNA content per cell.
